# Malignant mesothelioma in rare sites: two case reports

**DOI:** 10.3389/fonc.2025.1698949

**Published:** 2025-11-28

**Authors:** Qiguang Du, Jialu Guo, Zi Zhu, Yutong Zhang, He Cui, Zhongkai Xu, Rang Yang, Wenlong Liang

**Affiliations:** 1 Department of Breast Surgery, Second Affiliated Hospital of Harbin Medical University, Harbin, Heilongjiang, China; 2 Department of Oncology, Second Affiliated Hospital of Harbin Medical University, Harbin, Heilongjiang, China; 3 Department of Breast Surgery, Sixth Affiliated Hospital of Harbin Medical University, Harbin, Heilongjiang, China

**Keywords:** malignant, mesothelioma, pleura, peritoneum, immunotherapy, pathological diagnosis

## Abstract

Mesothelioma is a rare tumor originating from mesothelial cells in the pleura or other sites. Malignant pleural mesothelioma (MPM) is the most common aggressive type of mesothelioma (accounting for 81%) and carries a poor prognosis (median OS: 1 year). Diagnosing diverse histologic types is challenging. We report two cases: a 72-year-old female with peritoneal mesothelioma demonstrated a remarkable response, with the lesion shrinking from 150×160mm to radiologically undetectable after chemo-immunotherapy, resulting in a sustained complete response. Conversely, a 66-year-old male with MPM exhibited primary platinum resistance, progressing after first-line pemetrexed/lobaplatin and subsequent second-line chemotherapy. Peritoneal mesothelioma responded better to immuno-chemotherapy, while MPM showed platinum resistance. These contrasting disparate outcomes underscore that precise pathology and individualized treatment based on anatomic site are crucial, suggesting that malignant pleural and peritoneal mesothelioma may represent distinct clinical entities.

## 1 Introduction

Malignant mesothelioma is a rare and aggressive cancer arising from the mesothelial lining of serous cavities, predominantly the pleura and, less commonly, the peritoneum. It is strongly associated with asbestos exposure. With an annual incidence of approximately 1 per 100,000, it poses significant diagnostic and therapeutic challenges. Patients with MPM often present with symptoms such as pleural effusion, chest pain, and dyspnea, while peritoneal mesothelioma typically manifests with abdominal distension, pain, and ascites. Diagnosis relies on a combination of imaging and histopathological examination, with immunohistochemistry (IHC) playing a pivotal role in distinguishing it from metastatic adenocarcinoma. Despite advances, the prognosis remains poor, with a median overall survival for MPM of around 12 months. First-line treatment has historically been platinum-based chemotherapy, but the emergence of immune checkpoint inhibitors (ICIs) has recently altered the therapeutic landscape for selected patients. However, response to therapy is heterogeneous, and the biological drivers of this heterogeneity, particularly across different anatomical sites, are not fully understood. This knowledge gap underscores the need for detailed case reports that can generate hypotheses for future research. Herein, we report two cases of mesothelioma with different anatomical origins, combining dynamic imaging monitoring and changes in tumor markers to explore differences in diagnostic and therapeutic strategies.

## 2 Case presentation

### 2.1 Peritoneal mesothelioma case

Patient Information: A 72-year-old female, with a Karnofsky Performance Status (KPS) of 93.

Chief Complaint and Symptoms: The patient presented in February 2024 with lower abdominal distension and pain accompanied by anal pain.

Medical, Family, and Psychosocial History: The patient had a 6-year history of diabetes mellitus (managed with repaglinide), a 2-year history of depression (treated with escitalopram), and a 1-year history of bilateral lower extremity deep vein thrombosis (treated with rivaroxaban and aescin). No significant family history of genetic disorders or cancer was reported.

Relevant Physical Examination (at initial presentation): Physical examination revealed a well-nourished, conscious patient. The abdomen was flat, without surgical scars, palpable masses, tenderness, or rebound tenderness. Bowel sounds were normal. No edema was present in the lower extremities.

Relevant Past Interventions and Outcomes: In March 2024, an attempted surgical intervention at another hospital was aborted due to intraoperative hemodynamic instability (a sudden drop in blood pressure and tachycardia) under anesthesia. Subsequently, ultrasound-guided peritoneal fluid aspiration and pelvic mass biopsy were performed, which led to the definitive diagnosis.

### 2.2 Pathological assessment and rationale for immunotherapy

The biopsy specimen was analyzed at an external tertiary cancer center (Tianjin Medical University Cancer Hospital). The pathological diagnosis was malignant peritoneal mesothelioma (Pathology No. S2403930), confirmed by IHC showing positive staining for calretinin, WT-1, and D2-40. Critically, the IHC panel was negative for markers typically associated with ovarian carcinoma (e.g., PAX-8), effectively ruling out this common differential diagnosis. Unfortunately, the original pathological slides are not available for re-review or additional staining (e.g., for TILs, CD3, or PD-L1) due to the external institutional protocol. The decision to initiate combination immuno-chemotherapy was based on several factors (1): (1) the confirmed diagnosis of a lethal malignancy with high tumor burden; (2) the patient’s good performance status (KPS 93); (3) emerging, albeit preliminary, literature suggesting potential efficacy of ICIs in peritoneal mesothelioma; and (4) extensive discussion with the patient and family regarding potential benefits and risks, leading to informed consent.

### 2.3 Therapeutic intervention and follow-up

**Figure 1:**
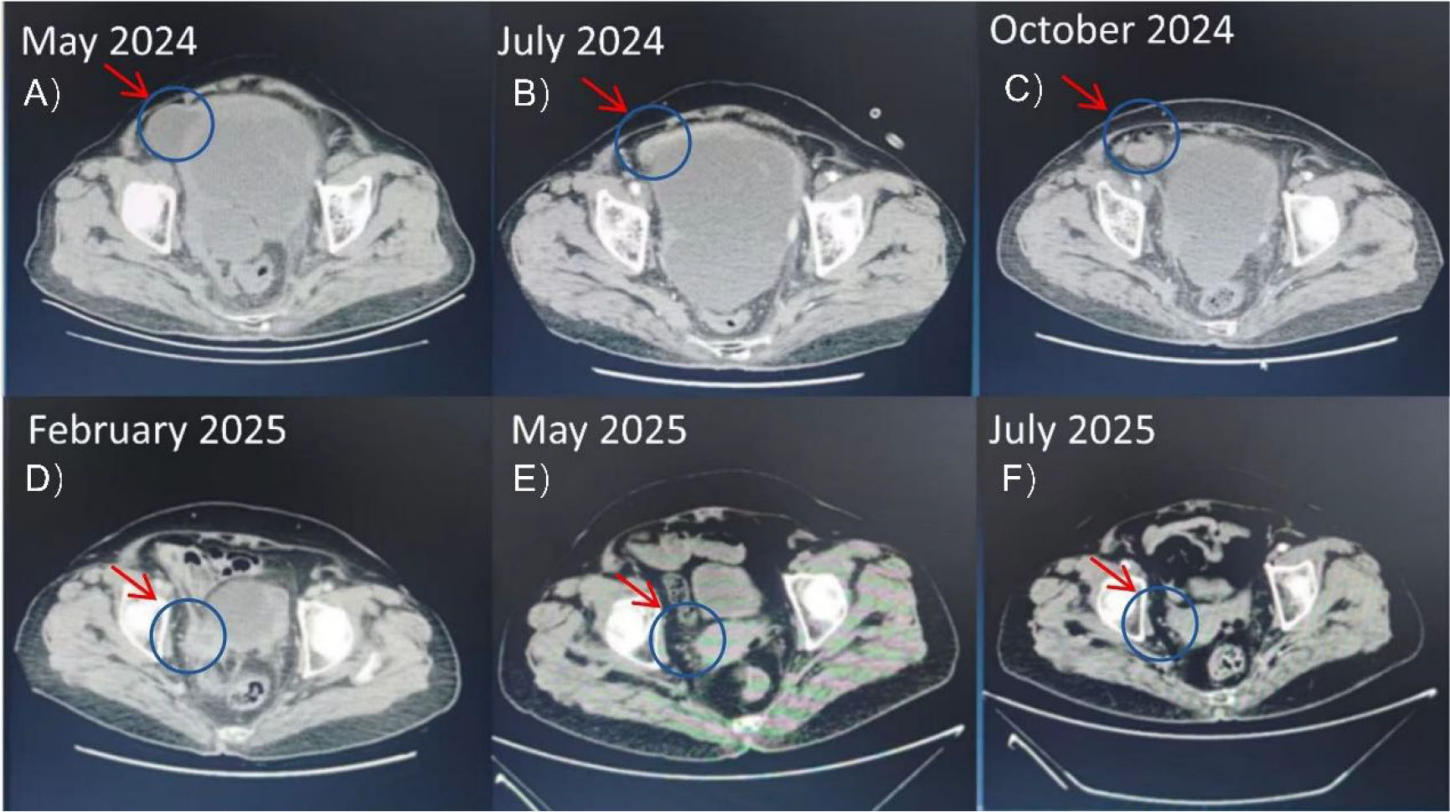
Serial axial CT scans of the pelvis in a patient with peritoneal mesothelioma. The arrows indicate the dominant pelvic mass. **(A)** Pre-treatment (May 2024) shows a large mass measuring 150 x 160 mm. **(B-F)** Subsequent images demonstrate a continuous reduction in tumor size following immuno-chemotherapy and maintenance immunotherapy, culminating in the resolution of the measurable lesion by July 2025. .

The following combination therapy regimen was administered at our hospital in May 2024, with each treatment cycle lasting 21 days: pemetrexed (500 mg/m^2^, day 1), cisplatin (75 mg/m^2^, day 1), and toripalimab (240 mg fixed-dose, day 1). After six treatment cycles, the pelvic lesions had shrunk compared to before, and symptoms of abdominal distension and pain had significantly improved. After six cycles of combination therapy concluding in September 2024, the patient achieved a significant partial response. Maintenance therapy with Toripalimab was initiated. As of the last follow-up in August 2025, the pelvic mass continued to shrink, and the patient remains in good clinical condition (**Figure 1**).

**Figure 2:**
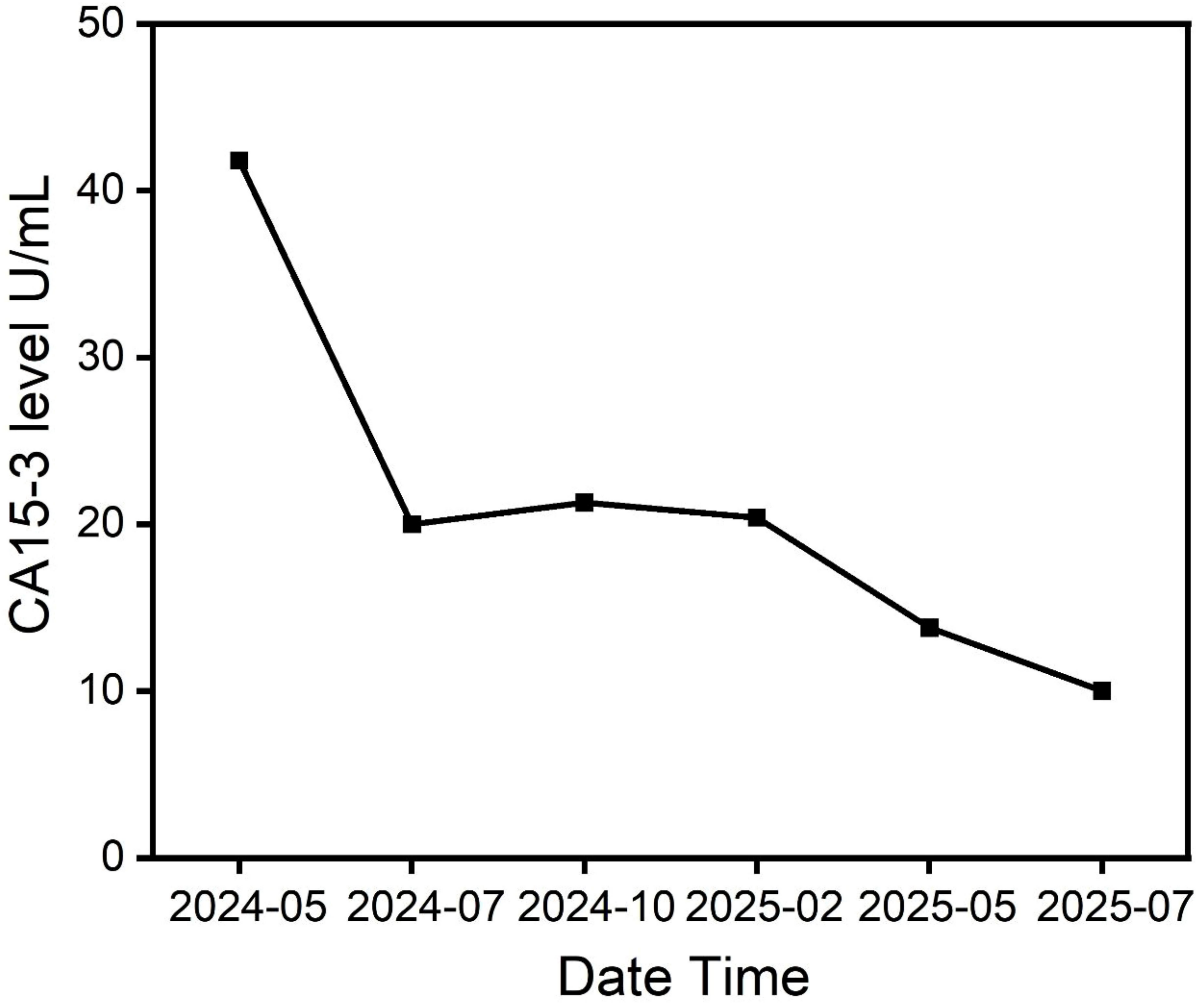
Serial changes in serum tumor marker CA15-3 (U/mL) in the patient with peritoneal mesothelioma. The decline in CA15–3 levels correlates with the radiographic tumor response observed in **Figure 1**.

In cases of peritoneal metastasis, we tested more commonly used markers (such as CA-125), but their baseline levels were not high. We chose to monitor CA15-3, although this marker is non-specific, because its baseline level was elevated and synchronized with changes in tumor burden. The tumor marker CA15–3 gradually decreased, indicating a significant therapeutic effect (**Figure 2**; **Table 1**).

**Table 1:** Timeline of diagnostic, therapeutic, and follow-up data for the peritoneal mesothelioma case.

Date	Diagnostic/intervention event	Key findings/outcomes
Feb-24	Initial Presentation	Lower abdominal distension and pain.
Feb-24	Pelvic MRI (Mudanjiang Hospital)	Multiple solid masses in the pelvis
May-24	Ultrasound, Biopsy & Pathological Diagnosis (Tianjin Cancer Hospital)	Confirmed diagnosis of Malignant Peritoneal Mesothelioma (Pathology No. S2403930).
May-24	Start of 1st Line Therapy	Toripalimab + Pemetrexed/Cisplatin (Cycle 1).
May-24	Baseline Assessment (CT & Tumor Marker)	Pelvic mass: 150 × 160 mm/CA15-3: 41.8 U/mL.
Jul-24	On-treatment Assessment (After 3 cycles)	Pelvic mass: 163 × 145 mm/CA15-3: 20.0 U/mL. Efficacy: Stable Disease (SD).
Sep-24	End of 1st Line Therapy (After 6 cycles)	Pelvic mass: 117 × 105 mm/CA15-3: 21.3 U/mL. Efficacy: Partial Response (PR).
Sep-24	Start of Maintenance Therapy	Toripalimab monotherapy initiated.
Feb-25	Follow-up	Pelvic mass: 67 × 27 mm/CA15-3: 20.4 U/mL. Efficacy: PR.
May-25	Follow-up	Pelvic mass: 41 × 25 mm/CA15-3: 13.8 U/mL. Efficacy: PR.
Jul-25	Follow-up	Pelvic mass: Undetectable/CA15-3: 10.0 U/mL. Efficacy: CR.

## 3 Case of MPM

Patient Information: A 66-year-old male.

Chief Complaint and Symptoms: Pleural effusion was discovered incidentally during a physical examination in May 2022. By September 2022, he developed unexplained chest tightness and wheezing, which progressed to dyspnea, cough, and sputum production by October 2022.

Medical, Family, and Psychosocial History: No past medical history, family history, or psychosocial history.

Relevant Physical Examination (at our institution post-diagnosis): The patient was in a fair general condition, conscious, and cooperative. The chest was symmetrical with normal respiratory movement. Lung auscultation revealed no dry or wet rales. The abdomen was flat, soft, and non-tender.

Relevant Past Interventions and Outcomes: Before diagnosis, approximately 2000ml of pleural effusion was drained for diagnostic purposes.

### 3.1 Pathological assessment and treatment rationale

Following histological and immunohistochemical analysis conducted by our hospital (Harbin Medical University Affiliated Hospital), the patient was diagnosed with mesothelioma in October 2022.The IHC profile was characteristic: CK5/6(+), Calretinin (+), WT-1(+), D2-40(+), with negative staining for TTF-1 and NapsinA, confirming the diagnosis and excluding lung adenocarcinoma. As this specimen was obtained through routine clinical diagnosis and treatment, supplementary analyses such as PD-L1 staining or next-generation sequencing (NGS) were not performed at the time, and the tissue block is no longer available for such subsequent studies. The initial first-line treatment regimen of pemetrexed/platinum-based drugs conformed to international guideline standards. Following disease progression, the choice of docetaxel/cisplatin was driven by the absence of standardized second-line regimens in international guidelines and limitations in access to therapies like immune checkpoint inhibitors (ICI) or bevacizumab. This approach aimed to utilize a different taxane and an alternative platinum agent while achieving efficacy and managing potential adverse reactions.

### 3.2 Therapeutic intervention and follow-up

**Figure 3:**
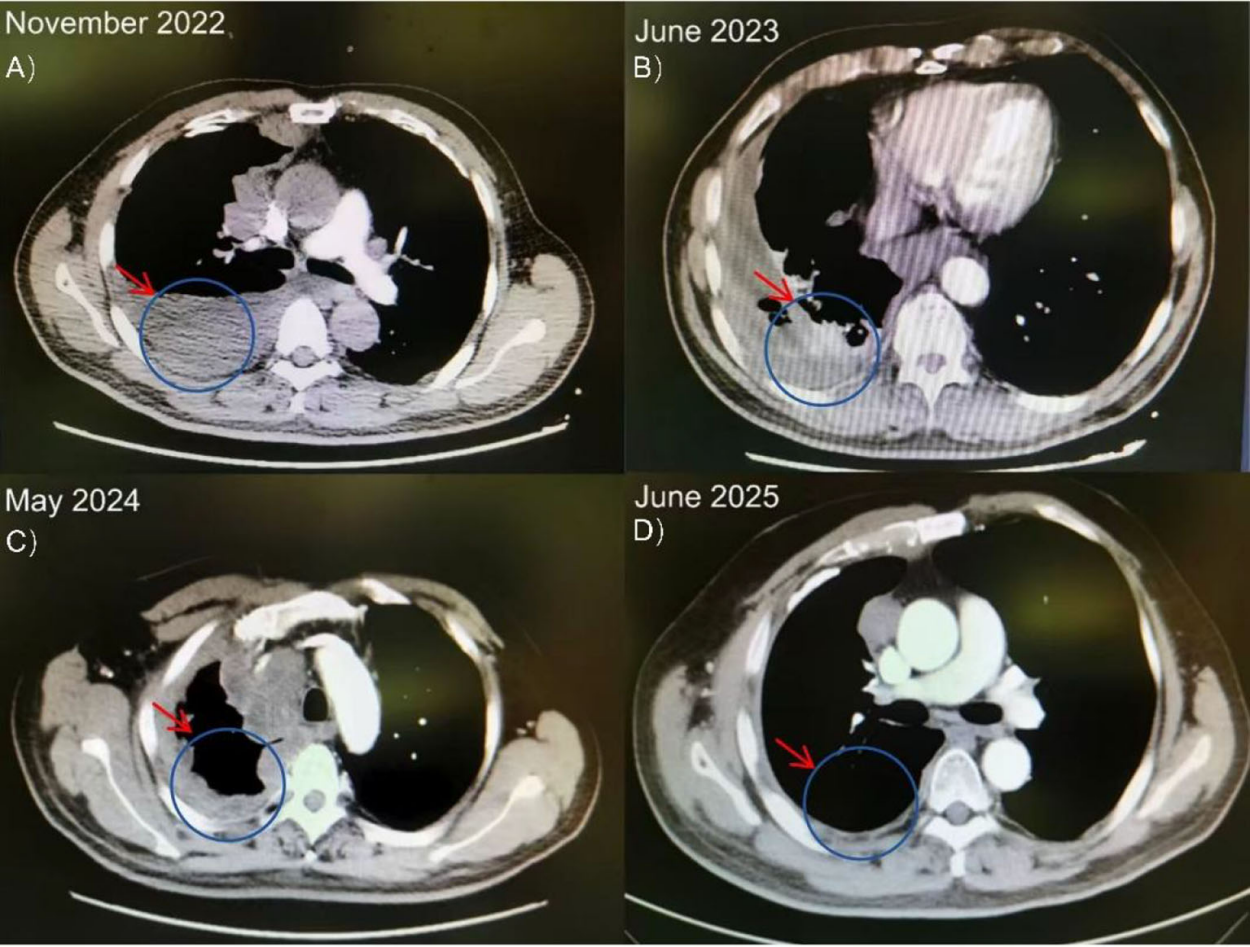
Serial axial chest CT scans of the patient with MPM. **(A, B)** Baseline imaging shows right pleural masses and effusion. **(C, D)** Subsequent scans demonstrate disease progression with the emergence of a new chest wall soft tissue mass (arrow) and increased pleural effusion despite second-line chemotherapy.

From January to June 2023, the patient received 8 cycles of first-line chemotherapy with pemetrexed (500 mg/m², Day 1) and lobaplatin (30 mg/m², Day 1) every 21 days. Post-treatment imaging revealed stable disease. However, due to significant chemotherapy-related toxicity, the regimen was subsequently de-escalated to pemetrexed monotherapy for 13 cycles. The patient eventually experienced disease progression, confirming primary platinum resistance. A contrast-enhanced chest CT in May 2024 showed a new soft tissue mass in the chest wall, prompting initiation of second-line docetaxel (75 mg/m², Day 1) and cisplatin (75 mg/m², Day 1). Despite this, follow-up imaging before submission revealed further disease progression, characterized by an increased right-sided pleural effusion and mediastinal lymph node enlargement (**Figure 3**; **Table 1**).

## 4 Discussion

This case analysis reveals the profound impact of anatomical location on clinical course and treatment outcomes by comparing malignant mesotheliomas originating from the peritoneum and pleura. The core finding demonstrates that despite identical histological subtypes, lesions originating from different anatomical sites yield markedly divergent treatment outcomes: one peritoneal mesothelioma achieved sustained complete remission following immunochemotherapy, whereas another pleural mesothelioma rapidly progressed after platinum-based chemotherapy. This stark contrast underscores that anatomical origin constitutes a critical, non-redundant factor determining disease biology, thereby influencing clinical presentation and therapeutic decision-making.

The diagnostic journey of both cases confirms that comprehensive immunohistochemical (IHC) analysis is crucial for accurate differential diagnosis. The initial diagnosis of peritoneal mesothelioma was misinterpreted as metastatic ovarian cancer. This was corrected through IHC testing, which confirmed the mesothelial cell lineage (calreticulin-positive, WT-1-positive, D2-40-positive) and excluded cancer markers (e.g., PAX-8-negative). Pleural mesothelioma, meanwhile, required differentiation from primary lung cancer with pleural metastasis. Ultimately, both cases relied on immunohistochemical markers for definitive diagnosis. This diagnostic precision is fundamental to selecting appropriate treatment strategies and underscores the necessity of multi-marker combined testing emphasized in the “Diagnosis and Treatment Guidelines for Malignant Mesothelioma (2)”.

We acknowledge several limitations in this study. The retrospective design restricted standardized biomarker analyses, such as the absence of tumor-infiltrating lymphocyte quantification, PD-L1 expression scoring, and next-generation sequencing, thereby limiting our interpretation of mechanisms underlying differential responses. Regarding efficacy assessment, we primarily relied on conventional imaging and serological markers, lacking more sensitive and accurate monitoring tools such as confirmatory biopsy or circulating tumor DNA testing. Despite these limitations, meaningful in-depth analysis remains feasible by integrating existing clinical data and literature. The first patient with peritoneal mesothelioma demonstrated an exceptional and durable response to pemetrexed/cisplatin combined with toripalimab, ultimately achieving radiographic complete remission (CR). Although not directly proven, this outstanding efficacy strongly suggests the tumor may possess an “immunologically sensitive” tumor microenvironment. Previous literature indicates that peritoneal mesothelioma more frequently exhibits a lymphocytic infiltration phenotype (3) and higher tumor mutational burden compared to pleural mesothelioma, characteristics potentially conferring greater sensitivity to immune checkpoint inhibitors. In stark contrast, the second pleural mesothelioma patient demonstrated primary resistance to first-line platinum-based chemotherapy and rapid progression on second-line therapy. This typical resistance pattern is often associated at the molecular level with mutations in genes such as BAP1, an immunosuppressive microenvironment, or low tumor mutational burden. Furthermore, in assessing treatment response, we primarily relied on dynamic monitoring via imaging (CT) and tumor markers (CA15-3). While these methods are gold standards in clinical practice, they lack histopathological reconfirmation or molecular-level monitoring of circulating tumor DNA (ctDNA). The latter can provide earlier and more sensitive information on treatment efficacy and resistance. Integrating these advanced monitoring tools into future clinical practice and research will facilitate more precise disease assessment.

Based on these observations, treatment strategies should prioritize immune checkpoint inhibitor plus chemotherapy for peritoneal mesothelioma due to its significant response (as demonstrated by this case’s progression-free survival >14 months). However, the rapid progression of malignant pleural mesothelioma (MPM) indicates the need to move beyond conventional chemotherapy frameworks. Following disease progression, molecular markers (e.g., BAP1 status) should be evaluated, and consideration should be given to anti-angiogenic agents (e.g., bevacizumab) or targeted therapies (per the 2025 Clinical Practice Guidelines for Histopathological Diagnosis of Mesothelioma). Its biological behavior appears determined by anatomical location. The intraperitoneal microenvironment, rich in immune cells, may enhance immunotherapy sensitivity, whereas the chronic inflammatory state and asbestos-fiber-induced genomic instability in the pleural cavity promote drug resistance. Although the two cases in this article are insufficient to establish a definitive treatment paradigm, they strongly suggest the future development of a stratified treatment approach based on anatomical location: peritoneal mesothelioma should be treated with immunotherapy combined with chemotherapy (based on our experience and growing literature), while pleural mesothelioma should integrate molecular subtype analysis from the outset of treatment (e.g., targeted therapy for BAP1-deficient patients) (4).

## 5 Conclusions

In summary, although malignant pleural mesothelioma and peritoneal mesothelioma share similar histological features, this study further confirms the notion that their distinct tumor microenvironments and molecular alterations are driven by their different anatomical locations. The starkly divergent clinical courses of the two patients suggest that anatomical location should be a key factor in patient risk stratification and treatment planning. Based on these observations, it warrants exploration whether malignant peritoneal mesothelioma may benefit more from immunochemotherapy, while malignant pleural mesothelioma requires early molecular subtyping. However, any anatomy-based stratified treatment approach remains in the preliminary exploratory phase. We urgently require larger, prospective clinical trials to systematically validate this anatomical stratification approach. The ultimate goal is to determine whether it can tangibly improve overall survival for all malignant mesothelioma patients and provide clinical practitioners with actionable guidance.

## Data Availability

The original contributions presented in the study are included in the article/supplementary material. Further inquiries can be directed to the corresponding authors.
